# Transcriptomics of the grape berry shrivel ripening disorder

**DOI:** 10.1007/s11103-019-00859-1

**Published:** 2019-04-02

**Authors:** Stefania Savoi, Jose Carlos Herrera, Astrid Forneck, Michaela Griesser

**Affiliations:** Division of Viticulture and Pomology, Department of Crop Sciences, University of Natural Resources and Life Sciences, Vienna, Konrad Lorenz Straße 24, 3430 Tulln, Austria

**Keywords:** RNA-sequencing, Switch genes, Sugars, Anthocyanins, Vitis vinifera, Zweigelt

## Abstract

**Key message:**

The lower expression at veraison of several ripening master regulators “switch genes” can play a central role in the induction of the berry shrivel ripening physiological disorder in grapevine.

**Abstract:**

Berry shrivel (BS) is a ripening physiological disorder affecting grape berry with visible symptoms appearing after veraison. Berry shrivel leads to shrinking berries with a reduced weight and a lower content of sugars and anthocyanins. In this study, for the first time a transcriptomic analysis coupled with selected metabolites quantification was undertaken to understand the metabolic modifications induced by the disorder. Different stages of berry development were considered including pre- and symptomatic berries. No metabolic alterations in the berry transcriptome and in the metabolite content was observed in pre-symptomatic and pre-veraison samples. Interestingly, at veraison, with still not visible symptoms appearing on the berry, a subset of genes, called *switch genes* previously suggested as master regulators of the ripening onset in grape berries, were strongly lower expressed in BS. Later during the ripening phase and with visible symptoms of the disorder, more than 3000 genes were differentially expressed. The genes up-regulated were related to hormone biosynthesis, response to stress and the phenylpropanoid pathway, while the genes down-regulated during ripening belonged mainly to the flavonoid pathway, and the sugar metabolism. In agreement, BS berries showed lower content of sugars and anthocyanins from the onset of veraison onward, while the amount of acids was not significantly affected. In conclusion, these results highlight a pivotal role of the switch genes in grapevine ripening, as well as their possible contribution to induce the ripening disorder berry shrivel, although it remains unclear whether this is part of the cause or consequences of the BS disorder.

**Electronic supplementary material:**

The online version of this article (10.1007/s11103-019-00859-1) contains supplementary material, which is available to authorized users.

## Introduction

Ripening physiological disorders, together with biotic and abiotic constrains, affect considerably the quality of the grape berry and the yield of the vine. Sunburn, late-season dehydration, bunch stem necrosis and sugar accumulation disorder are all classified as shriveling disorders and can commonly affect grapes. Nevertheless, some distinctive visible symptoms and morpho-anatomical characteristics and metabolites content make them distinguish although the etiology is still unclear (Krasnow et al. 2010; Bondada and Keller 2012b). Sunburn appears only on fruits exposed to the direct sun light; it is caused by high temperature and ultraviolet radiations and, depending on the severity of the stress, the consequences are browning spots on berry skin, cracking phenomena, and complete shrivel of the berry (Rustioni et al. 2014). Late season dehydration shrivels berries at almost the end of the ripening phase, leading berries with a reduced turgor and weight, but higher sugar content. There is no detectable physical or pathological damage to the berry skin, pedicel or bunch stem (Rogiers et al. 2006). Bunch stem necrosis is characterized by necrotic damage of the rachis followed by a reduction or cessation of assimilate transport towards berries. The effect on the berry metabolite content is in function of the timing when the necrotic lesions appear, and the altered metabolites composition influences the quality of the wines (Šuklje et al. 2016). Sugar accumulation disorder, also referred as simply berry shrivel (BS) and topic of this study, is characterized by berries with loss of turgor and weight, high acidity, low pH, low sugar concentration and, for red varieties low amount of anthocyanins (Krasnow et al. 2009; Griesser et al. 2012). BS symptoms appear after veraison when seeds are fully mature and therefore it does not interfere with the correct seed development (Bondada and Keller 2012a). Sugars mainly in the form of hexoses start to be accumulated in the vacuoles at the onset of veraison. Previous studies agree in a stopped sugar accumulation few days before symptoms became visible on the berry (Krasnow et al. 2009; Keller et al. 2016; Griesser et al. 2018). Malate and tartrate are the main organic acids present in the grape berry. It is reported that BS cause a slightly faster malate catabolism but has no significant effect on the final content of both tartaric and malic acid (Krasnow et al. 2009; Keller et al. 2016). BS differs from bunch stem necrosis because the latter starts with necrotic areas on the rachis and pedicel, and from the late season dehydration because the sugar content is low. Causes are still unknown and the lack of a reliable measurable parameter determining the onset of berry shriveling is one of the major difficulties to identify triggering factors.

The *V. vinifera* cv Zweigelt (St. Laurent × Blaufränkisch) is one of the most important red grape varieties in Austria. Several studies reported that this cultivar is particularly sensitive and highly affected by BS (in German Traubenwelke) (Knoll et al. 2010; Griesser et al. 2012; Bachteler et al. 2015; Griesser et al. 2017, 2018). Unsteady yields are problematic and lead to high economic losses for producers and winemakers since it is not possible to obtain good quality grape and wine from BS grape. BS affects not only Zweigelt, but also other cultivars in Europe and in USA such as Cabernet Sauvignon (Krasnow et al. 2009; Keller et al. 2016), Gewürztraminer, Pinot blanc and Pinot gris (Raifer et al. 2014) to cite a few.

Grapevine is a non-climacteric fleshy fruit and the fruit development follows a double sigmoid curve with three major phases (Coombe 1992). During ripening, berry genes expression and metabolism are finely orchestrated to change berry size, weight, and texture, to metabolize organic acids, and to accumulate numerous primary and secondary metabolites like sugars, pigments and aromas (Conde et al. 2007). Nevertheless, it is at the onset of veraison that in the berries there is a complete transcriptome reprogramming (Fasoli et al. 2012, 2018) and a small subset of genes, called switch, may actually be the key players driving this transition (Palumbo et al. 2014; Massonnet et al. 2017). The peculiarity of a switch gene is to be expressed in a low level during the immature/green phase of development, to switch on at the onset of ripening and being significantly induced and highly expressed during the mature/ripening phase. Moreover, switch genes are connected with several neighboring genes that display an opposite expression profile, i.e. they are expressed at high level during the immature/green phase, turn off at the onset of veraison and therefore expressed at low level during the mature/ripening phase. In the red-skinned grape varieties, 190 switch genes have been identified and 1266 associated negative neighboring connections (Palumbo et al. 2014). The main functional categories of the switch genes include *transcription factor activity*, *cell wall metabolism*, *development*, *response to stress*, *secondary metabolic process*, *DNA/RNA metabolic process*, and *carbohydrate metabolic process*; while the main functional categories of the neighboring genes include *photosynthesis*, *generations of precursors*, *response to endogenous stimuli*, *transcription*, *response to abiotic stimuli*, and *lipid metabolic process* (Palumbo et al. 2014).

In this study, for the first time a transcriptome analysis using the RNA-sequencing technique coupled with targeted metabolite analyses of major primary and secondary metabolites was performed comparing healthy and berry shrivel grape berries at six developmental stages including pre- and post-veraison stages and as well pre-symptomatic and symptomatic grapes. We hypothesize that transcript and metabolic changes in grapes at the onset of veraison might be involved in BS induction and symptoms development during the ripening process, narrowing the time frame window of BS induction. Our presented results support the idea of BS induction at the onset of veraison with anomalies in the transcriptomic reprogramming due to a disturbed or delayed shift of key ripening players, providing concise evidence on the time frame but leaving the question of the actual inducing factor still open.

## Materials and methods

### Plant material and berries sampling

Berries samples of the red cultivar Zweigelt grafted on Kober 5BB were collected in 2013 from a commercial vineyard located in Lower Austria (Antlasberg, Mailberg) with a recognized berry shrivel seasonal issue (Griesser et al. 2017). At the developmental stage of the grapes of pea-size (BBCH75), more than 300 grape clusters were randomly selected and labeled within the vineyard as reported from a previous year study (Griesser et al. 2017, 2018).

**Fig. 1:**
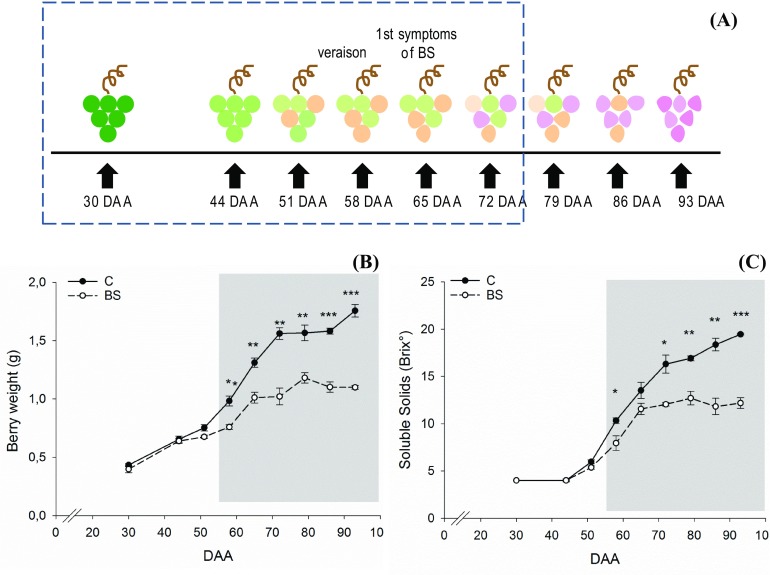
Outline of berry shrivel occurrence in 2013 season. **a** Graphical representation of the sampling; the data points in the dotted rectangle (30, 44, 51, 58, 65, and 72 DAA) were considered for transcriptomics and metabolomics analyses. Veraison and first visible symptoms of berry shrivel are indicated. Trend of the berry weight **b** and soluble solids **c** during berry development and ripening between control (“C)” and berry shrivel (“BS)” samples. Gray background highlights the onset of veraison in C vines. Bars represent mean values ± SE. Asterisks indicate significant differences between conditions evaluated by one-way ANOVA; *, **, *** indicates P < 0.05, P < 0.01 and 0.001 respectively

Soluble solids of all labeled grape clusters were weekly measured from BBCH77 to BBCH89, which corresponded to 30–93 days after anthesis (DAA), to follow berry growth and ripening. The distal part of the berry cluster (8 to 10 berries max, including pedicel and rachis) was weekly sampled and the berries were immediately frozen in liquid nitrogen in the field, transported to the lab, and stored at − 80 °C. Each labeled cluster was sampled only once during the season for two reasons: (i) do not interfere with the proper cluster development; (ii) to allow an acceptable a posteriori categorization in healthy grape or control (C) or berry shrivel (BS) samples. Samples selected for analyses were collected at 30, 44, 51, 58, 65, and 72 DAA. 50% veraison occurred approximately at 55 DAA. A schematic overview of the sampling timetable is shown in Fig. 1a. The posteriori categorization occurred at the end of the ripening phase combining soluble solids measurements and visual evaluation of the grape clusters as previously described (Keller et al. 2016; Griesser et al. 2017, 2018). For each sampling point and condition, three biological replicates were considered. Each biological replicate resulted from the pooling of three different bottom clusters and only samples which very clearly categorized into the categories “controls” and “BS” were used for further analyses. In case of Zweigelt, BS is affecting whole clusters therefore a reliable categorization of sampled bottom clusters throughout the season is possible. Frozen berries, without pedicel, were grinded to a fine powder under liquid nitrogen using a ball mill (Retsch MM400). The frozen powder was aliquoted for RNA and metabolites extraction as described below.

### Transcriptome analysis

Total RNA was extracted with the ‘Spectrum Plant total RNA’ kit (Sigma-Aldrich) from 0.2 g of frozen berry powder. The quantity and quality of the RNA were determined with a 2100 Bioanalyzer (Agilent Technologies). mRNA library preparation was performed with 1 μg of total RNA per sample using the TruSeq RNA Sample Prep Kit v2 according to the manufacturer’s instructions (Illumina). Sequencing was performed with an Illumina HiSeq 2500 platform at the Next Generation Sequencing (NGS) unit of the Vienna Biocenter Core Facilities GmbH (VBCF, Vienna, Austria).

An average of 21.9 M 50-nt single-end reads was generated per sample (Supplementary Table S1). Trimming for quality and length, were performed with Trimmomatic, version 0.36 (Bolger et al. 2014). Reads were aligned against the reference grapevine genome PN40024 12x (Jaillon et al. 2007), using the software Hisat2, version 2.1.0 (Kim et al. 2015). Aligned reads were counted with HTSeq-count (version 0.9.1) in intersection-non-empty mode for overlap resolution (Anders et al. 2015). Differentially expressed genes (DEG) (FDR < 0.05) analysis was performed with the R package DeSeq 2 (Love et al. 2014). To obtain a more informative list of genes a cutoff of log_2_FC |0.5| was applied. Functional annotations of genes were retrieved from (Grimplet et al. 2012). Principal component analysis (PCA) on the transcriptome dataset was performed and visualized using the R packages “stat” and “scatterplot3d” (Ligges and Mächler 2002). Gene ontology analyses were carried out using the complete list of DEG. Overrepresented genes categories were identified with the BINGO app 3.0.3 of Cytoscape 3.6.0 (Shannon et al. 2003; Maere et al. 2005) using GOSlim-Plant categories as described in (Savoi et al. 2016). Switch genes list was retrieved from (Palumbo et al. 2014) supplementary dataset 11. Heatmaps were drawn using R.

Quantitative real-time polymerase chain reaction (qPCR) analyses were carried out on a selected set of genes. The reverse transcription of RNA samples was performed with the QuantiTect Reverse Transcription Kit (Qiagen). qPCR cycling condition and calculation of the relative expression values were carried out as described in (Griesser et al. 2017, 2018).

### Metabolites analyses

Major sugars, organic acids, and anthocyanins metabolites were analyzed by HPLC (Dionex™ ICS-5000, Thermo Fischer Scientific) equipped with an electrochemical detector (pulsed amperometry) and a diode array detector (Dionex™ UltiMate™ 3000, Thermo Fischer Scientific). For the identification and quantification of the metabolites Chromeleon software, version 7.2, was used.

Major sugar compounds, without derivatization, and organic acids were extracted with the following procedure. In short, 1 g of frozen berry powder was dissolved in 10 mL of milliQ water, shaken for 20 min at room temperature, and centrifuge for 10 min. The surnatant was collected, properly diluted and filtered with 0.45 μm nylon syringe filter (Carl Roth).

For the sugars, the chromatography analysis was carried out using a Dionex CarboPac™ PA20 column, 3 × 150 mm coupled with a Dionex CarboPac™ PA20 guard column, 3 × 30 mm (Thermo Fischer Scientific) kept at 30 °C. Mobile phase A was milliQ water; mobile phase B was NaOH 100 mM (Thermo Fischer Scientific). The flow rate was 0.5 mL/min. The gradient profile was as follow: from 0 min to 11 min isocratic 52% B; followed by column cleaning, regeneration and back to the initial condition. Injection volume was 10 μL. During analysis samples were kept at 4 °C in the autosampler. Glucose, fructose and sucrose were identified and quantified using their reference standards. The concentration of each sugar was expressed as mg/berry grape.

For the organic acids, the chromatography analysis was carried out using an Acclaim™ OA column (Thermo Fischer Scientific), 5 μm particle size, 4 × 250 mm kept at 30 °C. Mobile phase was 100 mM Na_2_SO_4_, pH 2.65 with methanesulfonic acid. The flow rate was 0.6 mL/min. The gradient profile was isocratic with a run of 12 min. Injection volume was 10 μL. During analysis samples were kept at 20 °C in the autosampler to avoid tartaric acid precipitation. Organic acid compounds were detected at 210 nm and identified using their reference standards. The concentration of each compound was expressed as mg/berry grape.

Anthocyanins compounds were determined according to (Torres et al. 2017) with some modifications. In short, 1.8 mL of methanol:water 1:1 was added to 0.18 g of frozen berry powder. The metabolites were extracted using an ultrasonic bath for 1 h at room temperature; samples were then centrifuged for 10 min, the surnatant collected and filtered with 0.45 μm nylon syringe filter (Carl Roth). The separation was carried out using an Accucore C18 column, 2.6 μm particle size, 100 mm × 4.6 mm (Thermo Fischer Scientific) kept at 25 °C. Mobile phase A was milliQ water containing 10% of formic acid and mobile phase B was acetonitrile 100% HPLC grade (Chromasolv, Sigma-Aldrich). The flow rate was 0.368 mL/min. The gradient profile was as follow: 0 min 7.8% B; from 0.1 min to 14.1 min linear gradient to 30% B; from 14.1 min to 14.9 min linear gradient to 50% B; from 14.9 min to 16 min isocratic 50%B; from 16.1 min to 17.1 min back to the initial condition of 7.8% B. Injection volume was 10 μL. During analysis samples were kept at 4 °C in the autosampler. Anthocyanins compounds were detected at 520 nm and identified following the literature (Mattivi et al. 2006). The concentration of individual anthocyanin was expressed as standard of malvidin-3-glucoside (Extrasynthese) mg/berry grape.

### Statistical analysis

A one-way ANOVA was performed using JMP 7 (SAS Institute Inc.) to detect significant differences (*P *< 0.05) between C and BS samples at each data point. Graphs were drawn with Sigma Plot 13 (Systat Software Inc).

### Data availability

All raw transcriptomics reads have been deposited in NCBI Sequence Read Archive (http://www.ncbi.nlm.nih.gov/sra). The BioProject and SRA accession are PRJNA436693 and SRP134067, respectively.

## Results

### Berry growth and development

Berry shrivel affected the growth and the development of the berries. Among the grape clusters labeled at the beginning of the season, 36.5% developed BS symptoms during the ripening phase. The berries collected from shriveled samples and used for further analyses showed significantly reduced berry weight from the onset of veraison until berry ripening (Fig. 1b) and in the same timescale also soluble solids measured as a °Brix were significantly reduced (Fig. 1c).

### Berry shrivel transcriptome

We aimed to understand molecular changes induced by berry shriveling while possibly uncovering early triggering mechanisms before visible symptoms appears. Therefore, RNA sequencing was performed over six developmental stages, which corresponded to pre-veraison and early post-veraison sampling, and included four pre-symptomatic BS sampling (30, 44, 51, and 58 DAA) followed by two symptomatic BS sampling dates (65, and 72 DAA).

After trimming for length and quality, the average number of reads that uniquely mapped in the V1 version of the grapevine genome (Jaillon et al. 2007) was 17.2 M (Supplementary Table S1).

**Fig. 2:**
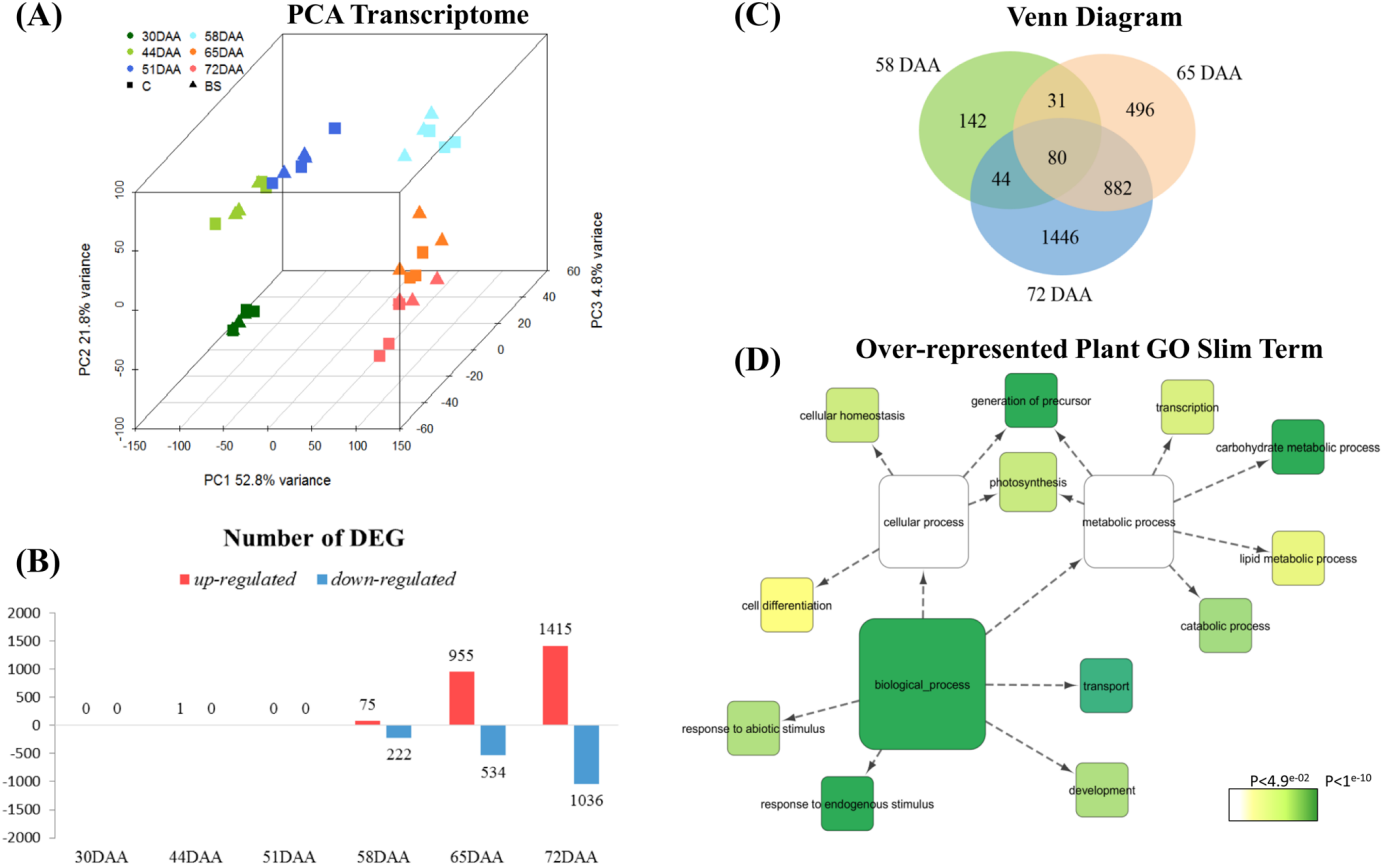
Analysis of the berry transcriptome in control (“C)” and berry shrivel (“BS)” samples. **a** Principal component analysis of the berry transcriptome of the 36 samples at 30, 44, 51, 58, 65, and 72 DAA. Squares and triangles represent C and BS sample, respectively. Different colors are for each single data point as reported in the legend. **b** Number of DEG at each data point; red and blue bars represent the up-regulated and down-regulated genes respectively. **c** Common and unique DEG at 58, 65, and 72 DAA are represented in the Venn diagram. **d** Plant gene ontology (GO) slim biological process categories enriched (P < 0.05) within the entire list of DEG

A principal component analysis (PCA) was performed to visualize the entire transcriptome trend of the 36 samples analyzed (2 conditions × 6 developmental stages × 3 biological replicates) (Fig. 2a). The first three principal components defined 52.8%, 21.8% and 4.8%, of the variance among samples, respectively. Berry transcriptome were distinctly separated in agreement to their developmental stage; however, within developmental stages, the separation between BS and C samples was less evident, with major degrees of separation in the last developmental stages.

The total number of differentially expressed genes (DEG) between the two conditions was 3122. The number of DEG in the three pre-veraison samples was 0, 1, and 0; while the number of DEG modulated by BS in post-veraison was 297 (75 up-regulated, 222 down-regulated) at 58 DAA, 1489 (955 up-regulated, 534 down-regulated) at 65 DAA and 2451 (1415 up-regulated, 1036 down-regulated) at 72 DAA (Fig. 2b, Supplementary Table S2). Some genes were differentially regulated in unison among two or three developmental stages and specifically 80 DEG were in common to the three ripening stages (Fig. 2c). Interestingly, among them, beside 13 genes with unknown function, there were 17 switch genes showing a persistent lower expression in BS berries over the ripening phases as compared to C samples, with only two exceptions. Twelve plant GO categories (slim biological processes) were significantly overrepresented among the DEG: *carbohydrate metabolic process*, *catabolic process*, *cell differentiation*, *cellular homeostasis*, *development*, *generation of precursors*, *lipid metabolic process*, *photosynthesis*, *response to abiotic stimulus*, *response to endogenous stimulus*, *transcription*, and *transport* (Fig. 2d).

Since the number of DEG in pre-veraison samples was extremely low using the current approach (DESeq 2, FDR < 0.05, and log2 FC |0.5|) we searched the DEG detected with the DESeq 2 method with only significance FDR < 0.05 regardless their expression but the results did not change. We therefore used the edgeR method (Robinson et al. 2010) with qCML approach, with GLM approach and with QL-FTest approach but also in this case the results did not change.

### Switch genes are lower expressed at the onset of ripening in BS samples

**Fig. 3:**
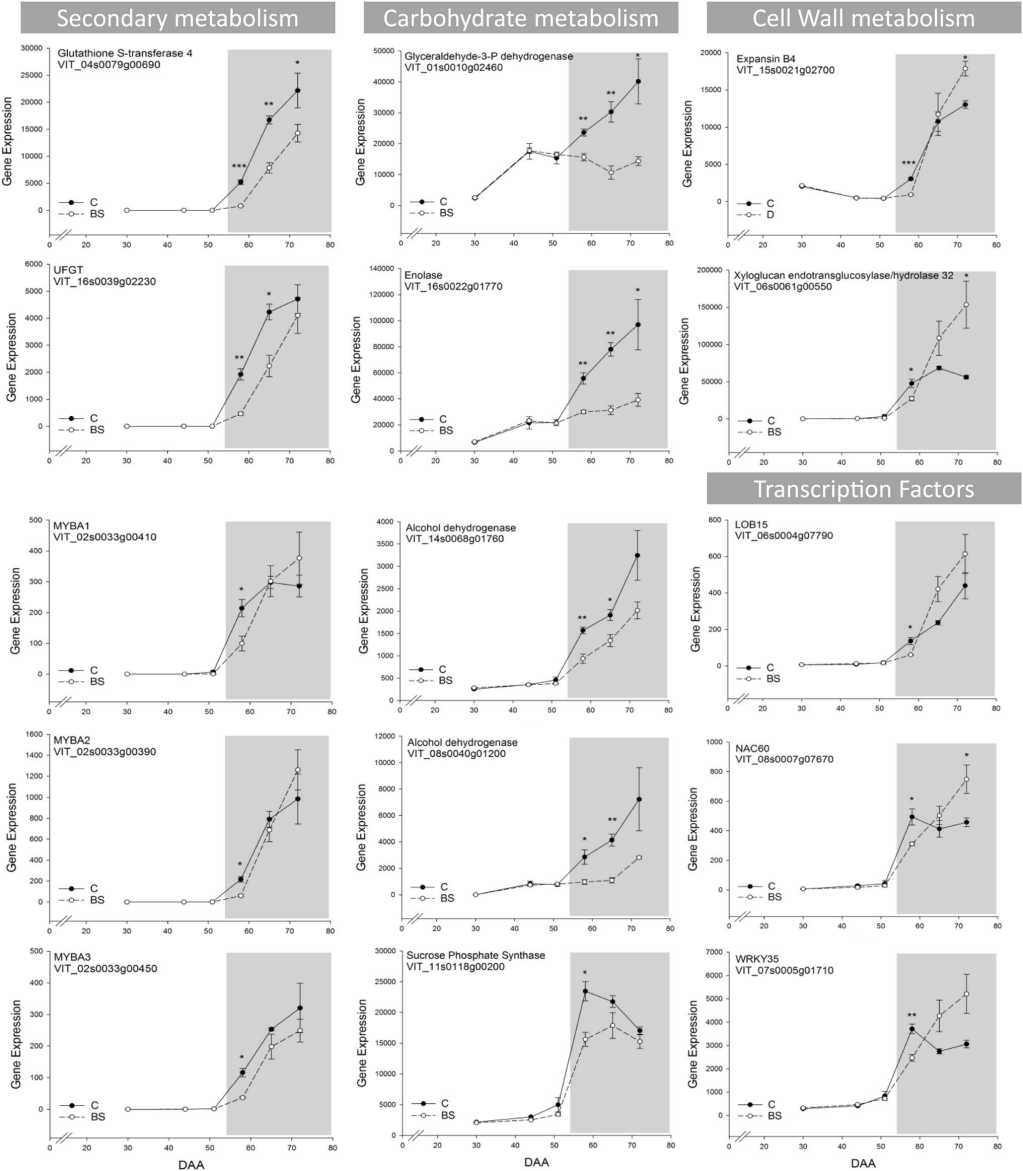
Expression profile of 15 selected switch genes. Gray background highlights the ripening stages from the onset of veraison. Bars represent mean values ± SE. Asterisks indicate significant differences between conditions *, **, indicates P < 0.05 and P < 0.01 respectively

**Fig. 4:**
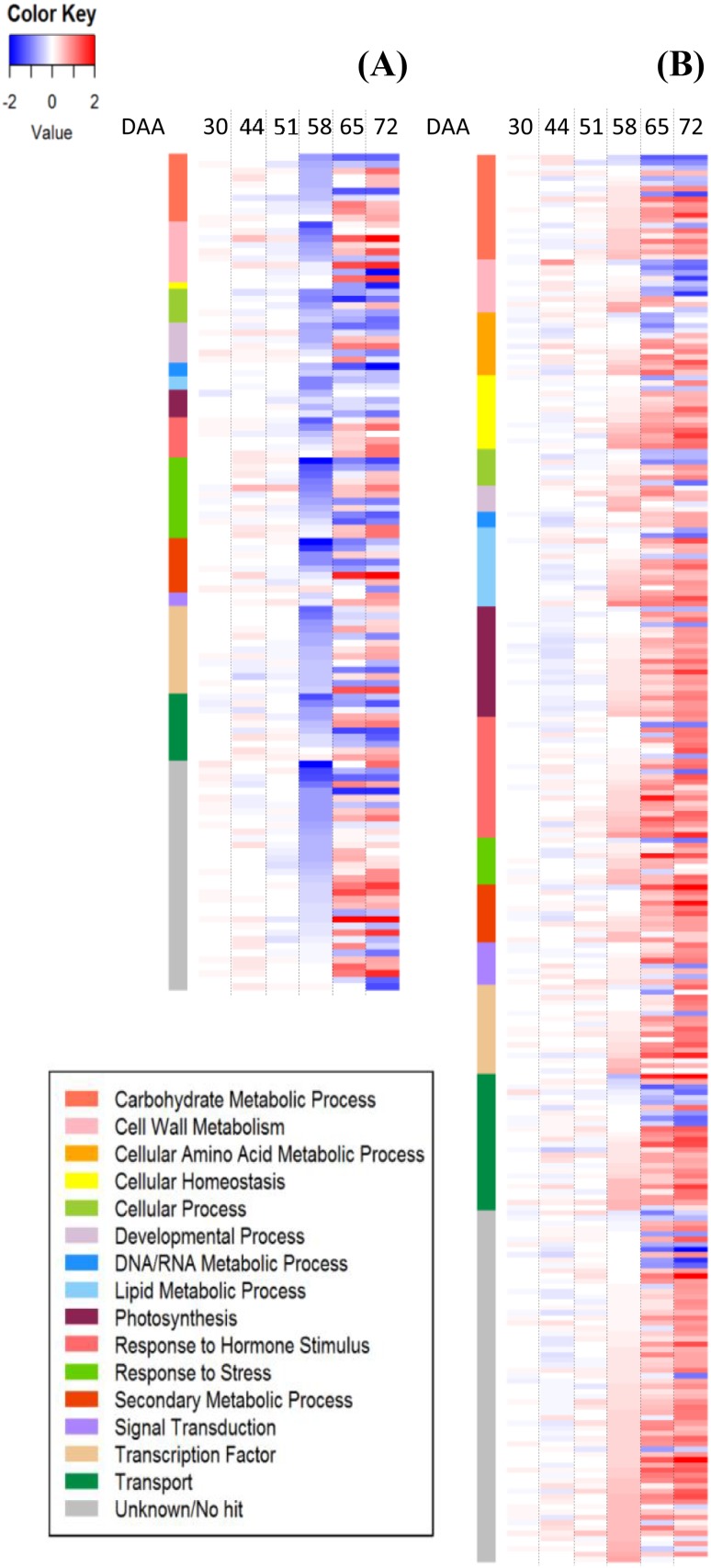
Complete list of Switch Genes (**a**) and Neighbor Gene (**b**) differentially expressed during the ripening berry phases are represented in heatmaps. Values are presented as the log_2_FC (BS/C) at 30, 44, 51, 58, 65, and 72 DAA. Blue and red colors indicate down- and up- regulation in BS, respectively. The colored side bar on the left indicates their biological process

Veraison is a crucial event in grape berry where many processes, like softening and important metabolites accumulation such as anthocyanins and aroma compounds start. During this key transition (58 DAA according to our sampling) we could identify 297 DEG (Fig. 2c). Most of them (75%) were down-regulated in BS. Among the 190 grapevine switch genes (Palumbo et al. 2014), at 58 DAA we identified 67 switch genes differentially expressed; all of them were down-regulated in BS samples. As switch genes, they were low expressed in pre-veraison (30, 44, and 51 DAA) and then they increased their expression value in post-veraison (58, 65, and 72 DAA) (Supplementary Table S3). During ripening (65 and 72 DAA), several switch genes showed a constant lower expression compared to C; others reached the same value of expression as in C with no significant differences, few exhibited a higher expression in particular at 72 DAA (Figs. 3 and 4). Most of these switch genes differentially expressed are known to play an important role during berry ripening (Fig. 3). For example, among them, there were genes related to the secondary metabolism process such as a glutathione-S-transferase 4 (*VviGST4*), and the UDP glucose:flavonoid 3-O-glucosyltransferase (*VviUFGT*); several transcription factors like *VviMYBA1*, *VviMYBA2* and *VviMYBA3*, a lateral organ boundaries domain protein (*VviLOB15)*, a NAC domain protein (*VviNAC60*), and a WRKY protein (*VviWRKY35*) Regarding the cell wall metabolism, there were an expansin B4 (*VviEXPB04*), and a xyloglucan endotransglucosylase/hydrolase 32 (*VviXTH32*). Other genes were related to the carbohydrate metabolism process such as an enolase, two alcohol dehydrogenases (*VviADH*), a glyceraldehyde-3-phosphate dehydrogenase (*VviGAPDH*) and a sucrose phosphate synthase (*VviSPS*). A complete list is available in Supplementary Table S3. Expanding the search to the other ripening stages of our study, there were in total 124 (65.3%) switch genes differentially expressed showing principally lower expression in BS compared to C with few exceptions.

### A closer examination of the genes differentially expressed during ripening

Remarkably, during ripening (65 and 72 DAA) and with manifested BS symptoms on the berry, we observed a higher number of DEG up-regulated (Supplementary Table S4). There were a conspicuous number of neighboring genes, which are negatively connected to the switch genes, displaying an opposite expression behavior, i.e. instead of being shut down at veraison they were up-regulated in BS during ripening (Fig. 4). Interestingly, many neighboring genes with higher expression in BS during the ripening phase were related to the photosynthesis and included genes encoding for proteins of the photosystem I and II, of the light-harvesting complex, and enzymes of the Calvin cycle such as phosphoribulokinase, ribulose-1,5-bisphophate carboxylase/oxygenase (*VviRuBisCO*) and sedoheptulose-1,7-bisphosphatase. Other genes up-regulated were genes related to hormone biosynthesis and signaling, mainly ethylene, auxin and abscisic acid. Concerning the ethylene, we observed up-regulation of two 1-aminocyclopropane-1-carboxylate oxidase (*VviACCoxidase*), genes that are deputed to the biosynthesis of ethylene, together with several signaling genes of the ERF/AP2 gene family; regarding the hormone auxin, there were up-regulated a tryptophan aminotransferase related (*VviTAR*) and three flavin monooxygenase like (*VviYUCCA*) which are auxin biosynthetic genes, five genes related to the indole-3-acetic acid homeostasis such as IAA-amido hydrolases and IAA-amido synthetases together with several ARF, Aux/IAA, and auxin response genes such as SAURs. Also, some abscisic acid related genes were up-regulated; for example, two protein phosphatases, a SnRK2, two abscisic acid receptor PYR/PYL/RCAR, and some ABA-responsive factors. Furthermore, some response to stress genes were up-regulated such as thaumatin, osmotin, and several dehydration-responsive protein; numerous genes up-regulated were involved in transport, in transcription factors activities (NAC, WRKY, MYB, bZIP, zinc finger), and genes of the phenylpropanoid pathway such as phenylalanine ammonia-lyase (*VviPAL*), 4-coumarate-CoA ligase (*Vvi4CL*), hydroxycinnamoyl-CoA:shikimate/quinate hydroxycinnamoyltransferase (*VviHCT*), caffeoyl-CoA acid 3-O-methyltransferase (*VviCCoAMT*), cinnamoyl CoA reductase (*VviCCR*) and ferulate 5-hydroxylase (*VviF5H*) and finally several thioredoxins and glutathione S-transferases (Supplementary Table S4).

On the other side, the genes down-regulated during ripening were genes related to glycolysis, TCA and flavonoid pathway (discussed below), genes involved in transport, some auxin signaling protein, but also an ethylene *VviACCoxidase,* even though its expression level was lower than the other isogenes up-regulated. Lastly, the genes related to the cell wall metabolism such as cellulases, pectinesterases, pectinacetylesterases, polygalacturonases, xyloglucan endotransglucosylase/hydrolase, endo-1,4-beta-glucanase and cellulose synthases were both up- and down-regulated by BS during ripening.

### Modulation of sugars and organic acids metabolism in BS berries

**Fig. 5:**
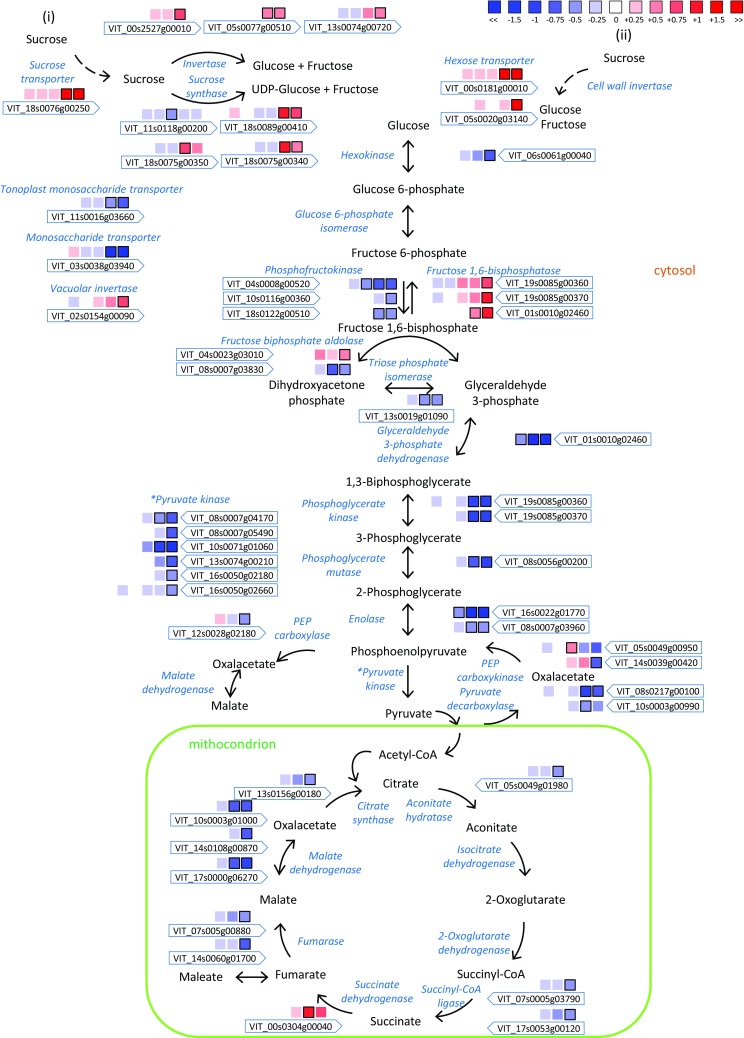
Impact of BS on the sugar metabolic pathway and TCA cycle. Log_2_FC (BS/C) values of DEG are presented at 30, 44, 51, 58, 65 and 72 DAA with series of boxes from left to right. Blue and red boxes indicate down- or up-regulation of the considered gene, respectively. Bold margins identify significant differences (P < 0.05) between control and berry shrivel samples

At veraison, besides berry softening, begins the accumulation of sugars in the vacuoles of the berries. BS berries are characterized by a lower amount of sugar therefore we explored the sugar related pathways. The DEG belonging to the sugar metabolism pathways as well the one of glycolysis and TCA in the grape berry are presented in Fig. 5 as the log_2_FC of BS compared to C. All data are presented in Supplementary Table S2 and S4.

Among the sugar transporters (Afoufa-Bastien et al. 2010), a sucrose transporter (*VviSUC27*) was up-regulated by BS at 65 and 72 DAA, as well as two hexose transporters (VviHT1-8 and VviHT5) that were up-regulated at 65 and 72 DAA and 72 DAA respectively. On the contrary a monosaccharide transporter (*VviMT*) and a tonoplast monosaccharide transporter (*VviTMT2*) were both lower expressed in BS at 65 and 72 DAA. Four sucrose phosphate synthases (*VviSPS*) were modulated by BS. Two of them were highly expressed in the berry, but one was down-regulated at 65 DAA, whereas the other was up-regulated at 65 and 72 DAA. Several neutral invertases (*VviNI*) were up-regulated by BS at 65 and 72 DAA. Furthermore, a vacuolar invertase, known as *VviGIN2*, was up-regulated at 72 DAA. Cell wall invertases (*VviCWI*) were not found to be differentially expressed by BS.

Due to differences in the response of these genes to BS showing lack of unison pattern of expression between classes and pathways, qPCR analyses were performed on six selected genes, namely a hexose transporter (*VviHT1*-*8*), a sucrose transporter (*VviSUC27*), a neutral invertase (*VviNI*), a vacuolar invertase (*VviGIN2*), a cell wall invertase (*VviCWI*), and a tonoplast monosaccharide transporter (*VviTMT2*) (Supplementary Fig. S2). The analysis confirmed the RNA-sequencing results matching the pattern of expression of these genes described above with genes up-, down-regulated or not affected by BS.

Glycolysis was strongly impaired in BS. Many genes such as one hexokinase, three 6-phosphofructokinases, one fructose-bisphosphate aldolase, one triose-phosphate isomerase, one glyceraldehyde-3-phosphate dehydrogenase, two phosphoglycerate kinases, one phosphoglycerate mutase, two enolases, and six pyruvate kinases were all down-regulated by BS. The only exception was a second fructose-bisphoshate aldolase which was up-regulated by BS at 72 DAA (Fig. 5).

Similarly, the TCA cycle was perturbed by BS with several genes down-regulated especially at 65 and or 72 DAA. Example of DEG were a citrate synthase, an aconitate hydratase, two succinyl-CoA ligases, two fumarases, and three mitochondrial malate dehydrogenases. The only exception was a succinate dehydrogenase that was up-regulated by BS at 65 DAA. Furthermore, phosphoenolpyruvate carboxylase which is involved in the first step of conversion of phosphoenolpyruvate in malate through oxaloacetate, was down-regulated by BS at 72 DAA (Fig. 5). On the contrary, the gene responsible for the rate-limiting step in the tartaric acid biosynthesis, the L-idonate dehydrogenase (DeBolt et al. 2006), was up-regulated at 65 and 72 DAA (Supplementary Table S4). Interestingly, this gene is one of the switch’s neighboring genes that is not turned off at veraison.

**Fig. 6:**
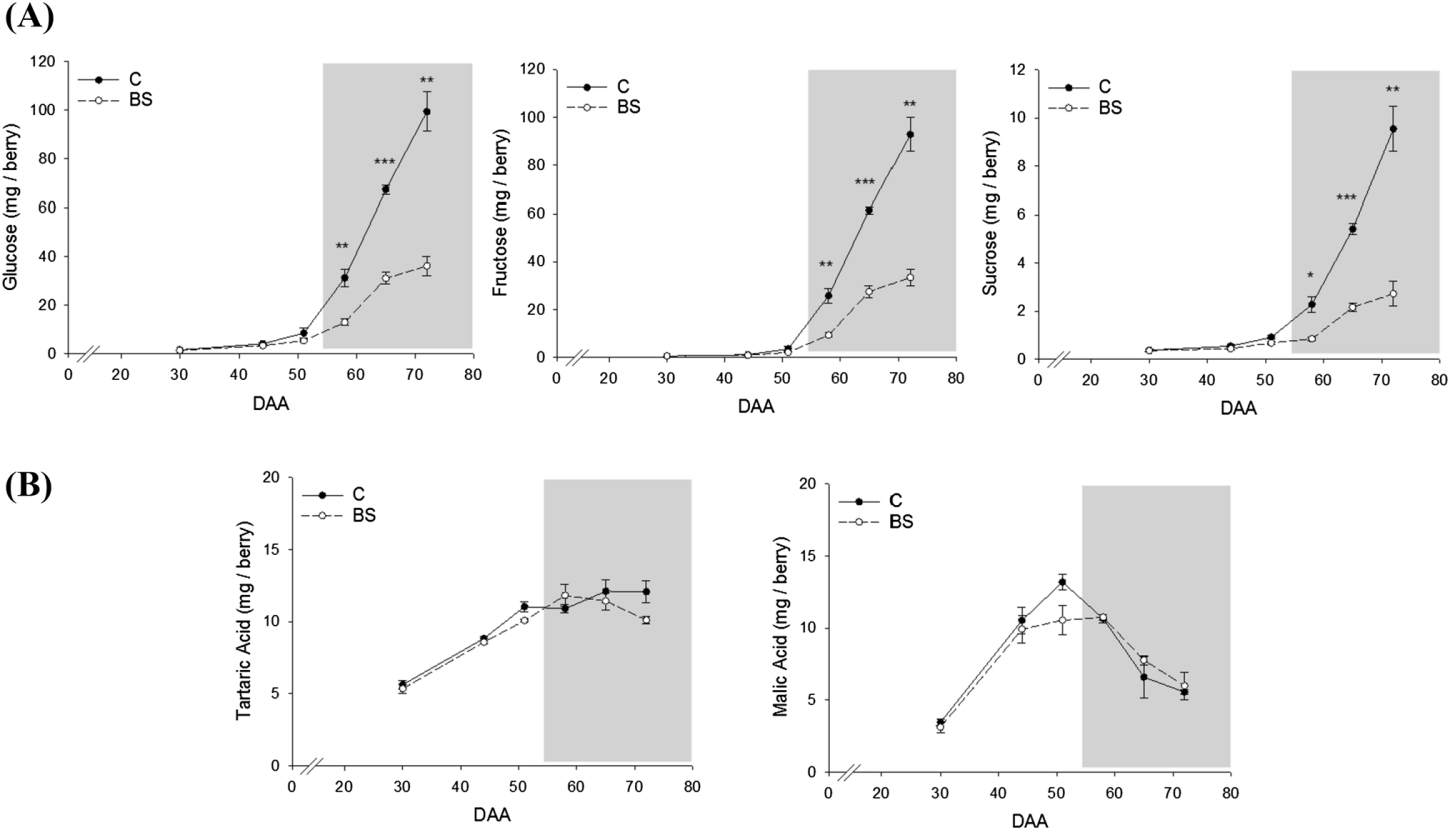
Profile of glucose, fructose, and sucrose content (**a**) and tartaric acid and malic acid content (**b**) during berry development and ripening expressed as mg per berry in C and BS samples. Gray background highlights the onset of veraison. Bars represent mean values ± SE. Asterisks indicate significant differences between conditions evaluated by one-way ANOVA, *, **, indicates P < 0.05 and P < 0.01 respectively

Targeted metabolite analyses were undertaken to correlate genes expression with sugar and organic acids content. We identified and quantified glucose, fructose, and sucrose, and tartaric and malic acids (Fig. 6). Significant differences in sugar accumulation were observed starting from the onset of veraison (58 DAA) and then during ripening (65 and 72 DAA), where the content of sugars in BS berries was significantly lower compared to the control. As described above, it is not possible to draw a clear pattern of the genes involved in the sugar metabolism but possibly less sugar is translocated in the vacuoles since a key *VviTMT* is reported to be down-regulated by BS. Conversely, the content of the two organic acids we measured did not show any significant differences between C and BS berries during the entire berry development and ripening. Malate and tartrate are accumulated in the berry before ripening and in our pre-veraison samples, with still no symptomatic berries, we did not observe any differences in the transcriptome of C and BS; therefore, the biosynthesis of these acids was not affected. Different is the situation of these acids during ripening where malic acid is catabolically consumed, while tartaric acid is not metabolized. No differences where observed in the malate catabolism during ripening between BS and control. On the contrary, one of the tartrate biosynthetic genes (L-idonate dehydrogenase) was up-regulated during ripening in BS, although no differences were observed in the final content of tartrate. A probable link between tartrate gene expression and metabolite content is the possibility that tartrate is used for the formation of esterified phenolic acids, like caftaric, coutaric, and fertaric acids as also several genes of the phenylpropanoid pathway were found up-regulated during ripening (Supplementary Table S4).

### The flavonoid pathway is hampered in BS with less content of anthocyanins

**Fig. 7:**
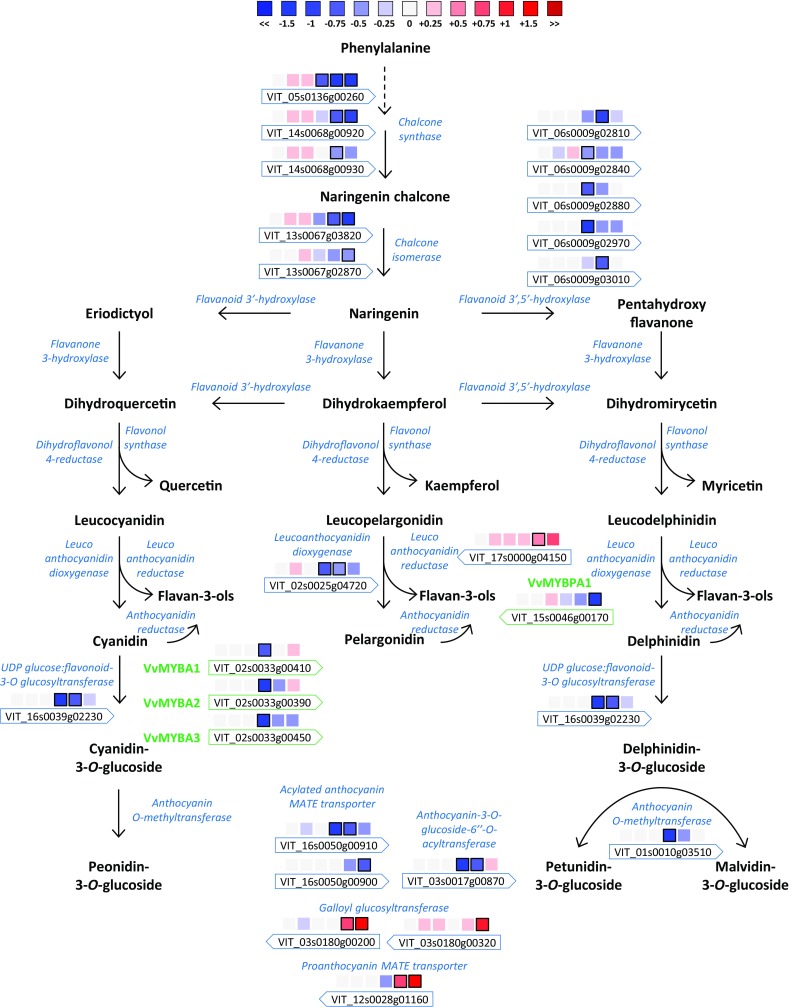
Impact of BS on the flavonoid pathway. Log_2_FC (BS/C) values of DEG are presented at 30, 44, 51, 58, 65 and 72 DAA with series of boxes from left to right. Blue and red boxes indicate down- or up-regulation of the considered gene, respectively. Bold margins identify significant differences between C and BS samples

Another berry shrivel symptom is a lower accumulation of anthocyanins. The effect of BS on the expression of the genes involved in the flavonoid metabolic pathway is expressed as the log_2_ fold change (log_2_FC) of BS compared to C; only the DEG were plotted into the metabolic pathways (Fig. 7, Supplementary Table S2, S4).

Berry shrivel modulated the expression of some genes that codify for structural enzymes of the flavonoid pathway. These genes were all down-regulated in BS after veraison, in one or in multiple ripening stages. The genes significantly lower expressed in BS were three chalcone synthases, two chalcone isomerases, five flavonoid-3′5′-hydroxylases and one leucoanthocyanidin dioxygenase (Sparvoli et al. 1994). Among the anthocyanin associated genes, the UFGT, one anthocyanin-*O*-methyltransferases (Fournier-Level et al. 2011), one anthocyanin-3-*O*-glucoside-6′’-*O*-acyltransferase (Rinaldo et al. 2015), and two acylated anthocyanin MATE transporters (Gomez et al. 2009) were down-regulated by BS after veraison. Furthermore, as mentioned before, the transcription factors regulating anthocyanins biosynthesis (*VviMYBA1*-*2*-*3*) were all down-regulated at 58 DAA.

The only upregulated genes were belonging to the branch leading to flavan-3-ols and proanthocyanidin biosynthesis. A leucoanthocyanidin reductase was up-regulated by BS at 65 DAA; two glycosyltransferases contributing in the galloylation of proanthocyanidins (Khater et al. 2012) were both up-regulated at 65 and 72 DAA and 72 DAA, respectively, as well as the proanthocyanidins transporter (Pérez-Díaz et al. 2014) that was up-regulated at 65 and 72 DAA, even though the associate transcription factor (*VviMYBPA1*) was down-regulated at 72 DAA.

**Fig. 8:**
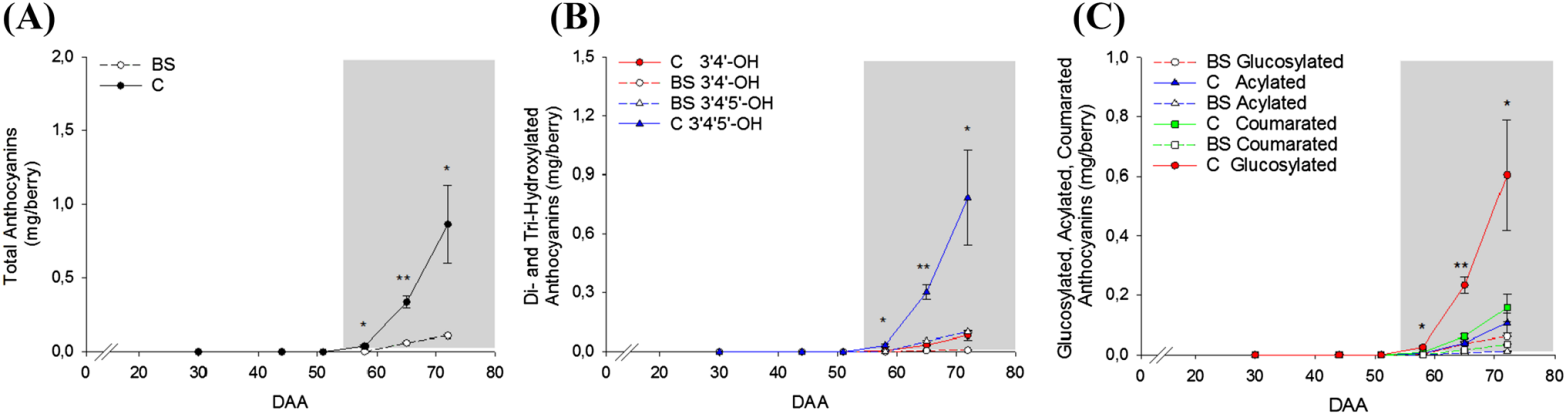
Profile of **a** total anthocyanins content, **b** di-hydroxylated (3′4′-OH) and tri-hydroxylated (3′4′5′-OH) anthocyanins content, and **c** glucosylated, acylated, and coumarated anthocyanins content in C and BS samples expressed as mg per berry basis. Gray background highlights the onset of veraison. Bars represent ± SE. Asterisks indicate significant differences between conditions evaluated by one-way ANOVA *, **, indicates P < 0.05 and P < 0.01 respectively

Fifteen anthocyanins were identified and quantified in a target metabolite analysis undertaken at each developmental stage, considering three biological replicates per condition, to correlate genes expression with the anthocyanins profile. The accumulation content profile of each of them in BS and C grape berries during development is reported in Supplementary Fig. S1. Cyanidin, peonidin, delphinidin, petunidin, and malvidin in the form 3-*O*-glucosilated, acetylated and *p*-coumarated were detected. Figure 8 reports the total content of anthocyanins (a), the content of di- and tri- hydroxylated anthocyanins (b), and their partitioning in forms (c) as mg/berry. Before veraison (30, 44, and 51 DAA) anthocyanins were not detected; their accumulation started at the onset of ripening (58 DAA) with higher predominance of tri-hydroxylated anthocyanins. Total content of anthocyanins was significantly lower in BS samples during berry ripening. In general, glucosylated anthocyanin were the main compounds present (68.3% of the total content) followed by the coumarated one (19.8%) and finally the acylated (12.0%). Among the five anthocyanins, malvidin was the most abundant in all the three forms. In this case, the lower content of anthocyanins in BS berry well correlates with the down-regulation of the entire flavonoid pathway.

## Discussion

Currently the triggering factors leading to berry shrivel disorder in grapevine and its related symptoms such as reduced sugars accumulation, low level of anthocyanins and flaccid and shrinking berries, visible few days after veraison, remain unknown.

Past studies focused on morpho-anatomical issues (Hall et al. 2011; Bondada and Keller 2012a; Bondada 2014; Crespo-Martínez et al. 2019), nutritional aspects (Bachteler et al. 2015; Bondada 2016; Griesser et al. 2017), viticultural practices (Raifer et al. 2014) or on a restricted list of genes (Griesser et al. 2018).

Here, for the first time, a comprehensive RNA-sequencing analysis was performed, coupled with basic metabolites analyses on healthy and berry shrivel berry samples collected at six different developmental stages.

The first three samplings were collected before veraison, when the symptoms of the disorder were not visible yet. The transcriptomics analysis revealed no differences in term of genes expression between the two conditions suggesting that all the berries were following a normal and proper development during the first growing phase and that the disorder was not induced so far. Previous studies regarding the seed development, which is a pre-veraison process, did not find any difference in seed weight, shape, color, and viability (Bondada and Keller 2012a) when comparing healthy and BS berries.

Differentially expressed genes (more than 3000 genes) were instead observed after veraison.

Firstly, the modulation of sugar import and accumulation once the grape berry became a valuable sink organ is a crucial aspect in BS berries. In grapevine, carbohydrates in the form of sucrose are transported from the leaves (source) to the berries (sink) through the phloem and apoplastically unloaded (Zhang et al. 2006). Sucrose must then be cleaved by invertases or sucrose synthases in the two hexoses, glucose and fructose, before being transported and stored in the vacuoles. Our results did not show a clear pattern of expression as some isoforms of these multigenic families were up-regulated (i.e. sucrose and hexose transporters, neutral invertases), other down-regulated (i.e. sucrose phosphate synthase, tonoplast monosaccharide transporters), or not affected (i.e. a vacuolar invertase and the cell wall invertases) by BS. Similar pattern of expression of these genes was already reported in a previous study (Griesser et al. 2018) with samples collected in a different season. As shown in Fig. 6a, glucose, fructose and sucrose were accumulated in lower amount in BS berries compared to C starting at veraison. Nevertheless, some sugars were accumulated in the BS berries indicating that the process is not completely interrupted but possibly less sugars is translocated and accumulated in the vacuoles. In fact, a tonoplast monosaccharide transporter known as TMT2 was downregulated in our dataset. This gene is known to be highly expressed at veraison and be one of the responsible for sugar vacuolar accumulation at the onset of ripening (Terrier et al. 2005; Lecourieux et al. 2014). Besides the biochemistry related processes during ripening, a possible additional explanation of lower sugar accumulation could be a disturbed translocation of assimilates within the vascular system of affected grape clusters. Previous studies showed reduced cell viability in the rachis of BS grapes possibly leading to a reduced transport function, although obvious necrosis on the outside of the rachis is not observed in our case (Hall et al. 2011; Bondada 2016). Recently, in-depth microscopic study of symptomatic BS Zweigelt grapes showed cell deformations and signs of total or more likely partial occlusion of the phloem sieve elements (Crespo-Martínez et al. 2019). A drawback of all anatomical studies so far is that pre-symptomatic analyses were not possible, as up to date no method exists to predict BS clusters in vineyards as well as a reliable method to induce BS. The question of causes and consequences is therefore crucial to understand BS. It seems evident that BS is induced around veraison (this study) or just before veraison (Griesser et al. 2017, 2018) and anatomical studies or phloem flow analyses at that time point are needed. A complete metabolic shutdown of berry metabolism is not observed due to BS, therefore a certain support of the vascular system seems to be sustained otherwise bunch stem necrosis symptoms could be the consequence.

Secondly, major perturbations were observed in the genes belonging to the flavonoid pathway where many of them were lower expressed in BS samples especially after veraison and in particular chalcone synthases and isomerases, flavonoid-3′5′-hydroxylases, *VviUFGT* and *VviMYBA1*-*2*. The only exception was the up-regulation of the branch leading to the flavan-3-ols production (*VviLAR*). Accordingly, the anthocyanins analysis confirmed a lower content of all the 15 anthocyanins in BS berries from veraison onward. This result is in agreement with (Griesser et al. 2018) and it suggests an unusual shutdown of this crucial pathway after veraison. Besides, sugars and anthocyanins accumulations are tightly interconnected; through sugar sensing and signaling, the anthocyanin biosynthesis is induced and therefore the anthocyanins content increases alongside sugars accumulation (Vitrac et al. 2000; Zheng et al. 2009; Lecourieux et al. 2014).

But it is at the onset of veraison, with still no visible symptoms of the disorder on the berries, that we observed the first transcriptomics differences between the two conditions, with many genes differentially expressed in BS samples. In particular, several switch genes, that are considered master regulators of the ripening process in grapevine, (Palumbo et al. 2014; Massonnet et al. 2017) were lower expressed in BS. The failure of such genes to switch at veraison increasing their expression at levels comparable to control affected numerous other genes and lastly the entire ripening phase. Among the DEGs, several of these master regulators of ripening that were lower expressed in BS are important genes involved in the carbohydrate metabolism, secondary metabolism, but also cell wall metabolism, response to stress, and transcription factors.

In grapevine, there is a cascade of events that triggers berry ripening (Fasoli et al. 2018) and several of these events depend on one or multiple switch genes functions. After the berry transcriptome reprogramming induced by the switch genes with the suppression of the vegetative pathways, the decrease in cell turgor (i.e. softening) and the concomitant increase in abscisic acid (ABA) are one of the earliest event during the onset of ripening (Castellarin et al. 2015). ABA has been recognized as the triggering signal of ripening (Wheeler et al. 2009), inducing sugar accumulation and anthocyanins biosynthesis (Gambetta et al. 2010; Castellarin et al. 2015). All these early processes were affected in our study. We did not observe the suppression of the vegetative pathways since, for example, many genes related with photosynthesis were still highly expressed in BS during ripening when normally the transcript abundance of most of them decline with increasing sugar levels in the berries (Ghan et al. 2017; Fasoli et al. 2018). Regarding berry softening and therefore the cell wall metabolism, an expansin of the group B (*VviEXPB4*), highly expressed in the berry, was down-regulated at veraison together with a xyloglucan endotransglycosylases. In plants, expansins and xyloglucan endotransglycosylases are the primary catalysts of the controlled cell wall loosening required for cell expansion and softening (Kaewthai et al. 2013; Dal Santo et al. 2013). Additionally, at the onset of veraison, there is a multifaceted hormonal signal crosstalk. In particular, in absence of a significant ethylene burst in non-climacteric fleshy fruits such as the grape, the auxin level decrement (Gouthu and Deluc 2015; Vondras et al. 2016) and the ABA increment are the events and the hormones that promotes the ripening process (Kuhn et al. 2014; Fortes et al. 2015). Recently, (Pilati et al. 2017) focused on the signaling network of ABA at the onset of veraison, which consists of ABA metabolism (biosynthesis, degradation, conjugation, and transport), perception, signaling cascade and extended network. There are no switch genes (in grapevine in general) involved in the ABA metabolism such as NCEDs, ABA 80-hydroxylases, ABA glucosidases, or ABC transporters, or genes related to the ABA perception such as the PYL/PYR/RCAR family receptors, PP2C phosphatases, and kinases such as SnRK2, CPK, and MAPK. Nevertheless, several ABA modulated genes in the extended network (Pilati et al. 2017) and also various transcription factors belonging to the ABA signal cascade (NACs, MYBs, AP2/ERFs), listed in this case as switch genes, were lower expressed by BS in our dataset at the onset of ripening (58 DAA). TFs have fundamental role in regulating many biological processes including development, ripening, signaling, and biotic and abiotic responses by controlling target genes expression. Unlike tomato, the model climacteric fleshy fruits where the transcription factors RIN, NOR, CNR, FUL1, FUL2, TAGL1, HB1 and AP2a together with ethylene signaling regulate the ripening phase in response to a developmental switch (Karlova et al. 2014), there is still limited information on the specific TFs involved in the grape ripening program. In our dataset a NAC60, a WRKY35, a LOB15, AP2/ERFs and Zinc Fingers were lower expressed in BS samples at the onset of veraison, while during the following ripening phases their expression increased. Lastly, as we described above and shown in Figs. 5 and 7, both the carbohydrate metabolism and the secondary metabolism in particular the flavonoid pathway with the branch related to anthocyanins were affected by BS with a reduced sugar accumulation and anthocyanins production.

During ripening (65 and 72 DAA) we observed a higher number of DEG up-regulated. Interestingly, there were genes related to hormones, stress related genes, transcription factors and the activation of the phenylpropanoid pathway. These transcriptomics changes resembled the changes described for late stages of berry over-ripening (Cramer et al. 2014) with more emphasis on the ones happening during the post-harvest withering process (Zamboni et al. 2008; Rizzini et al. 2009; Zenoni et al. 2016). In both cases an up-regulation of ethylene genes was reported suggesting a role of this hormone in the perception of stress due to fruit senescence and dehydration. About dehydration, ABA is not only the ripening hormone, but has a central role in drought stress through the activation of the ABA-dependent and ABA-independent signaling pathways (Savoi et al. 2017) with the transcriptional activation of several transcription factors of the ERF/AP2, ARF, NAC, WRKY gene families as we reported up-regulated by BS. The modulation of the phenylpropanoid pathway, also this already reported in withering berries (Zenoni et al. 2016), fits with a higher content of hydroxycinnamic acids, as reported for example for caftaric acid in Griesser et al. (2018) and can possibly explain the use of tartaric acids necessary for their esterification.

In conclusion, the post-veraison alteration in term of gene expression and in the metabolite content of sugars and anthocyanins are not the causes leading to the berry shrivel but they are consequences of a disrupted inception of the entire ripening process which brings to the berry shrivel physiological disorder. We highlighted that this might be due to a lower expression of several switch genes that do not allow an appropriate reprogramming of the berries from the vegetative to the ripening program. Nevertheless, further studies are necessary to define the reasons why *switch genes* do not *switch* at veraison at expression levels comparable to control and if other players are involved. New evidences in tomato support the idea of the involvement of micro-RNA (miRNA) and/or long non-coding RNA (lcnRNA) in regulating fruit ripening through TFs modulation (Chen et al. 2015; Li et al. 2018). Both miRNA and lcnRNA families are known in grape (Mica et al. 2010; Vitulo et al. 2014) nevertheless up to date there are no studies about their role or expression during grape berry development and ripening but only a recent study addressing UV-B effects on miRNAs in the post-transcriptional regulation of TFs (Sunitha et al. 2019).

## Electronic supplementary material

Below is the link to the electronic supplementary material.

Supplementary material 1 (PDF 153 kb). **Fig. S1** Profile content of single anthocyanins in control (C) and berry shrivel (BS) berries during fruit ripening. Data are expressed as mg/berry

Supplementary material 2 (PDF 23 kb). **Fig. S2** qPCR relative gene expression analysis of six selected genes in control (C) and berry shrivel (BS) during fruit ripening. Gene expression level analyzed with RNA-sequencing is reported in inset graphs for comparison. Bars represent ± SE. Asterisks indicate significant differences between treatments at P < 0.05 (*)

Supplementary material 3 (DOCX 20 kb). **Table S1** RNA sequencing analysis metrics. Transcriptome analyses were performed in C and BS berries at six selected berry developmental stages (30, 44, 51, 58, 65, and 72 DAA) using an Illumina HiSeq platform

Supplementary material 4 (XLSX 202 kb). **Table S2** Summary of differentially expressed genes and associated information (12xV1 identification number, log_2_FC (BS/C) and adjusted P value) identified at 44 (a), 58 (b), 65 (c), and 72 (d) days after anthesis (DAA)

Supplementary material 5 (XLSX 132 kb). **Table S3** List of differentially expressed “switch genes” (a) and “neighbor gene” (b) and associated information (12xV1 identification number, predicted functional annotation, log_2_FC (BS/C), adjusted P value at the six developmental stages, and average level of expression at 30, 44, 58, 65, and 72 DAA in C and BS)

Supplementary material 6 (XLSX 948 kb). **Table S4** List of differentially expressed genes (a) up-regulated and (b) down-regulated during ripening (65 and 72 DAA) and associated information (12xV1 identification number, predicted functional annotation, major functional category, log_2_FC (BS/C), adjusted P value at the six developmental stages, and average level of expression at 30, 44, 58, 65, and 72 DAA in C and BS)
