# Supplementary material for: Transcriptomics of the grape berry shrivel ripening disorder
Source: Plant Mol Biol. 2019 Apr 2;100(3):285–301. doi: 10.1007/s11103-019-00859-1 (PMC6542784; doi:10.1007/s11103-019-00859-1)

Content of Glucosylated Anthocyanins

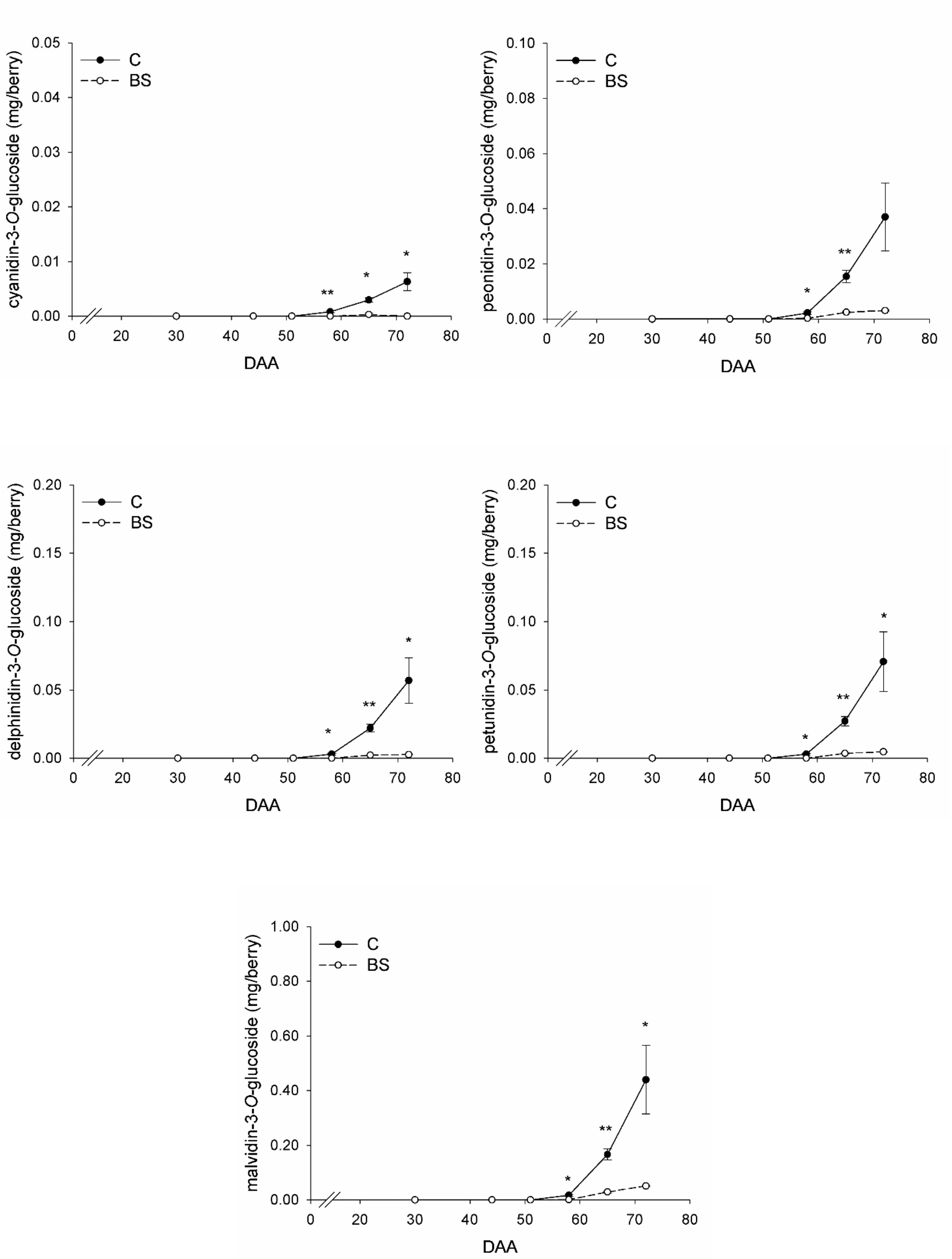

Content of Acylated Anthocyanins

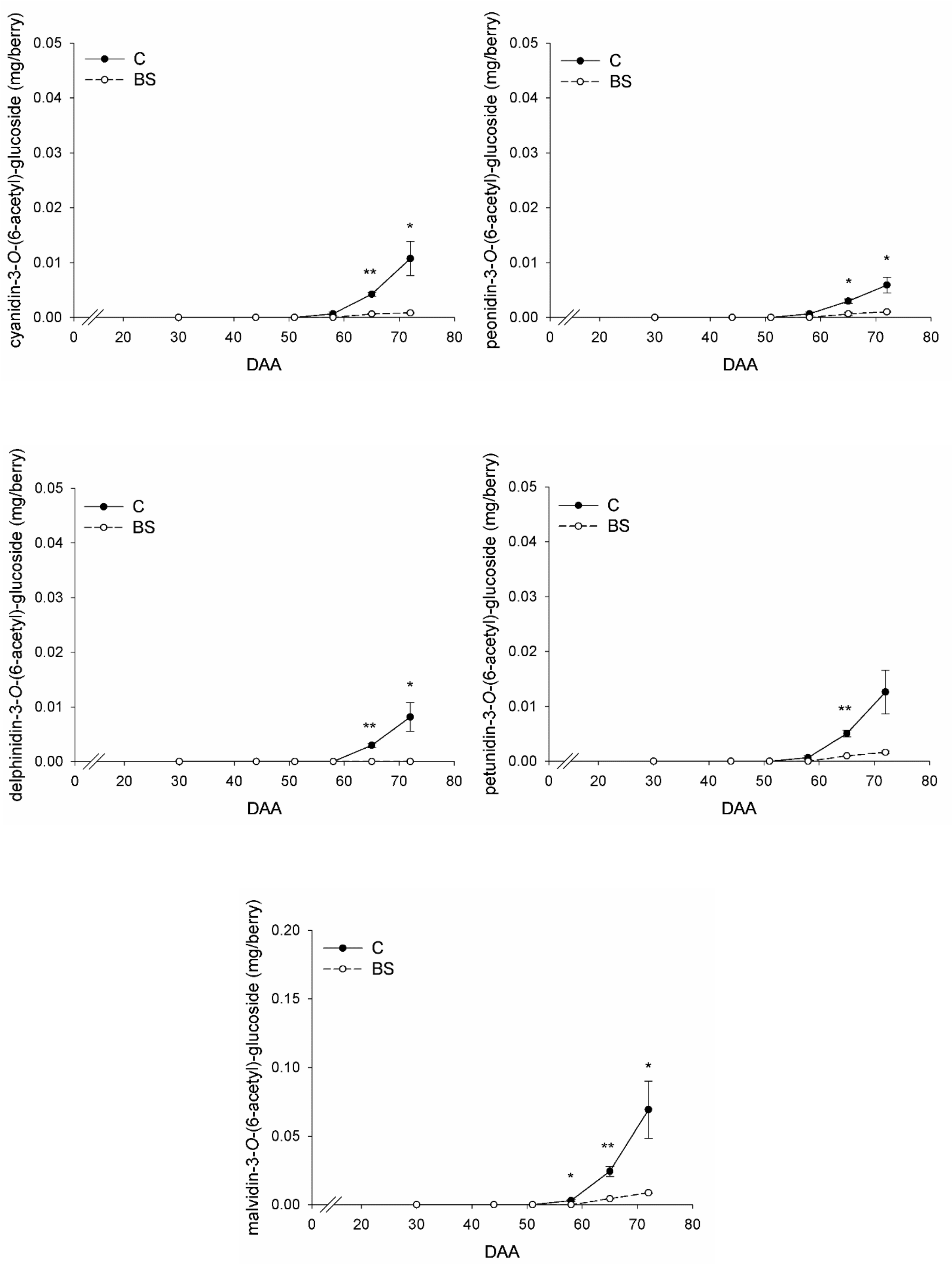

Content of Coumarated Anthocyanins

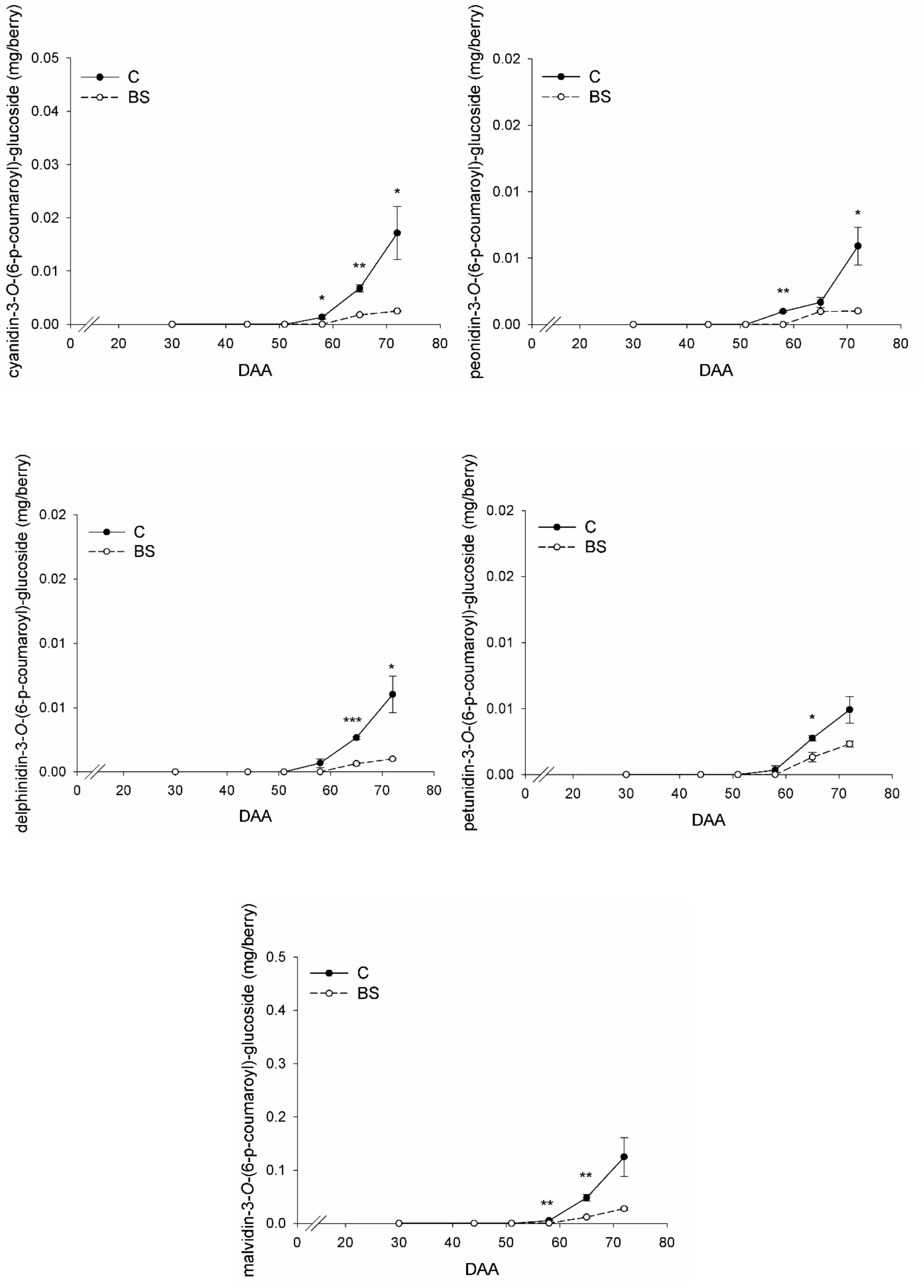

Supplement: Supplementary file 1 — Supplementary material 1 (PDF 153 kb). Fig. S1 Profile content of single anthocyanins in control (C) and berry shrivel (BS) berries during fruit ripening. Data are expressed as mg/berry [file 11103_2019_859_MOESM1_ESM.pdf]
