# Supplementary figures and images for: Transcriptomics of the grape berry shrivel ripening disorder
Source: Plant Mol Biol. 2019 Apr 2;100(3):285–301. doi: 10.1007/s11103-019-00859-1 (PMC6542784; doi:10.1007/s11103-019-00859-1)

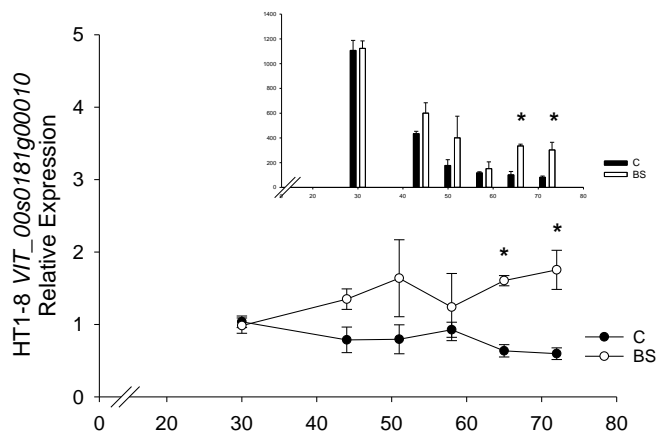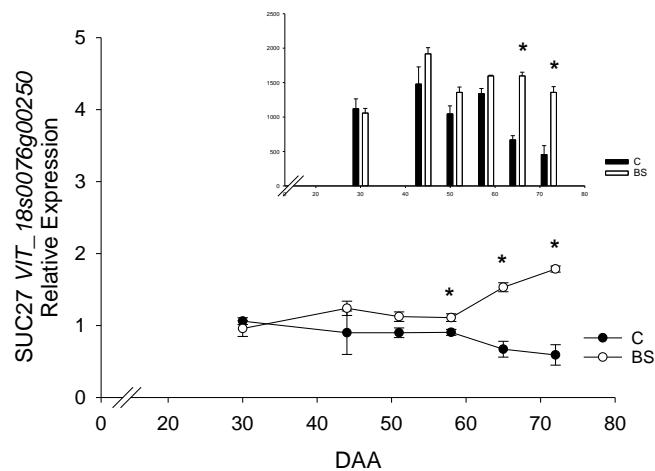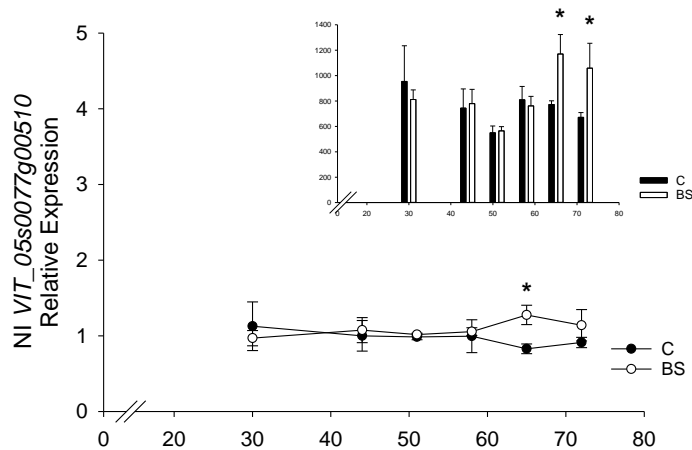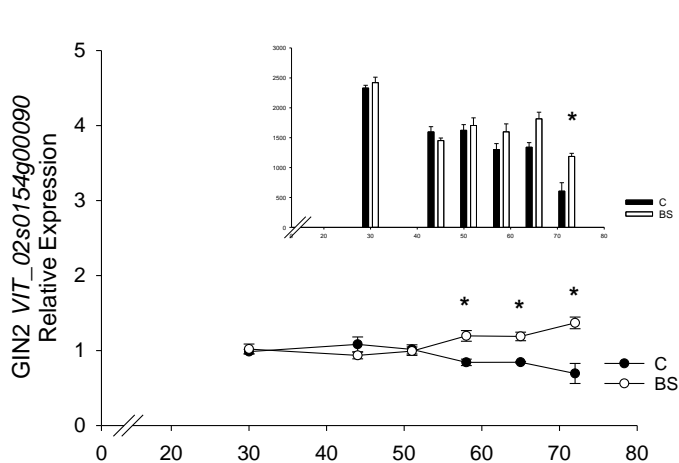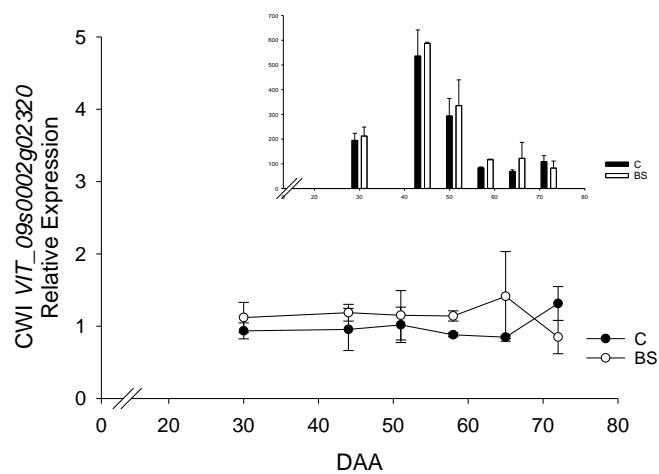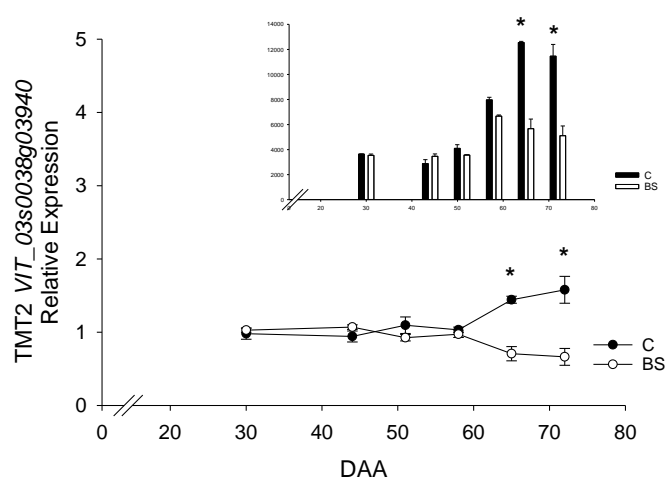

Supplement: Supplementary file 2 — Supplementary material 2 (PDF 23 kb). Fig. S2 qPCR relative gene expression analysis of six selected genes in control (C) and berry shrivel (BS) during fruit ripening. Gene expression level analyzed with RNA-sequencing is reported in inset graphs for comparison. Bars represent ± SE. Asterisks indicate significant differences between treatments at P < 0.05 (*) [file 11103_2019_859_MOESM2_ESM.pdf]
