# Supplementary material for: Transcriptomics of the grape berry shrivel ripening disorder
Source: Plant Mol Biol. 2019 Apr 2;100(3):285–301. doi: 10.1007/s11103-019-00859-1 (PMC6542784; doi:10.1007/s11103-019-00859-1)
Supplement: Supplementary file 3 — Supplementary material 3 (DOCX 20 kb). Table S1 RNA sequencing analysis metrics. Transcriptome analyses were performed in C and BS berries at six selected berry developmental stages (30, 44, 51, 58, 65, and 72 DAA) using an Illumina HiSeq platform [file 11103_2019_859_MOESM3_ESM.docx]

| **Table S1.** RNA sequencing analysis metrics. Transcriptome analyses were performed in C and BS berries at six selected berry developmental stages (30, 44, 51, 58, 65, and 72 DAA) using an Illumina HiSeq platform. | | | | | | | |
| --- | --- | --- | --- | --- | --- | --- | --- |
| **Sampling**  **(DAA)** | **Treatment** | **Biol. Rep.** | **Sequenced**  **Reads** | **Filtered Reads** | **Mapped**  **Reads** | **Unique**  **Reads** |  |
| 30 | C | 1 | 21,920,570 | 21,663,964 | 20,083,625 | 17,592,420 |  |
| 30 | C | 2 | 21,489,156 | 21,240,987 | 20,059,363 | 17,662,210 |  |
| 30 | C | 3 | 21,400,289 | 21,145,364 | 19,945,544 | 17,540,143 |  |
| 30 | BS | 1 | 26,864,118 | 26,548,585 | 25,184,040 | 22,437,626 |  |
| 30 | BS | 2 | 21,688,524 | 21,434,919 | 20,187,161 | 17,824,797 |  |
| 30 | BS | 3 | 27,657,022 | 27,333,138 | 25,234,444 | 21,866,807 |  |
| 44 | C | 1 | 18,333,386 | 18,118,193 | 15,940,319 | 13,130,130 |  |
| 44 | C | 2 | 19,034,531 | 18,812,311 | 16,489,548 | 13,524,684 |  |
| 44 | C | 3 | 21,036,635 | 20,800,663 | 18,114,488 | 14,772,694 |  |
| 44 | BS | 1 | 16,809,306 | 16,619,547 | 15,628,387 | 13,720,389 |  |
| 44 | BS | 2 | 31,949,229 | 31,584,530 | 25,574,741 | 21,357,506 |  |
| 44 | BS | 3 | 23,555,131 | 23,288,195 | 21,740,155 | 18,805,804 |  |
| 51 | C | 1 | 20,880,869 | 20,625,426 | 19,444,423 | 17,132,153 |  |
| 51 | C | 2 | 20,557,630 | 20,314,593 | 19,254,700 | 17,087,431 |  |
| 51 | C | 3 | 23,398,750 | 23,116,087 | 21,938,368 | 19,423,097 |  |
| 51 | BS | 1 | 25,216,239 | 24,920,856 | 23,642,166 | 21,295,594 |  |
| 51 | BS | 2 | 25,299,107 | 24,993,653 | 23,793,610 | 21,022,595 |  |
| 51 | BS | 3 | 15,449,427 | 15,264,048 | 14,428,982 | 12,754,866 |  |
| 58 | C | 1 | 14,245,265 | 14,137,739 | 13,317,664 | 11,883,365 |  |
| 58 | C | 2 | 21,764,240 | 21,604,535 | 20,492,876 | 18,332,049 |  |
| 58 | C | 3 | 19,018,237 | 18,873,128 | 17,934,602 | 16,058,886 |  |
| 58 | BS | 1 | 22,914,227 | 22,743,856 | 21,584,428 | 19,252,338 |  |
| 58 | BS | 2 | 20,951,473 | 20,792,242 | 19,563,171 | 17,291,525 |  |
| 58 | BS | 3 | 22,647,701 | 22,484,179 | 21,126,352 | 18,823,634 |  |
| 65 | C | 1 | 22,991,609 | 22,716,108 | 21,446,166 | 18,687,235 |  |
| 65 | C | 2 | 22,227,464 | 21,958,408 | 20,710,460 | 18,102,749 |  |
| 65 | C | 3 | 21,854,225 | 21,591,766 | 20,405,972 | 17,795,697 |  |
| 65 | BS | 1 | 25,689,387 | 25,371,269 | 23,464,330 | 20,071,572 |  |
| 65 | BS | 2 | 25,672,091 | 25,361,568 | 23,873,108 | 20,815,402 |  |
| 65 | BS | 3 | 18,828,712 | 18,449,339 | 17,211,057 | 14,983,456 |  |
| 72 | C | 1 | 22,403,654 | 22,224,920 | 21,088,716 | 18,525,828 |  |
| 72 | C | 2 | 18,171,185 | 18,028,822 | 16,929,975 | 14,689,728 |  |
| 72 | C | 3 | 22,328,688 | 22,164,495 | 20,890,606 | 18,116,003 |  |
| 72 | BS | 1 | 17,763,287 | 17,620,291 | 16,509,113 | 14,327,185 |  |
| 72 | BS | 2 | 13,265,120 | 13,163,056 | 12,336,109 | 10,757,978 |  |
| 72 | BS | 3 | 14,407,691 | 14,297,603 | 13,322,642 | 11,481,060 |  |
